# A Recurrent De Novo Terminal Duplication of 14q32 in Korean Siblings Associated with Developmental Delay and Intellectual Disability, Growth Retardation, Facial Dysmorphism, and Cerebral Infarction: A Case Report and Literature Review

**DOI:** 10.3390/genes12091388

**Published:** 2021-09-07

**Authors:** Ji Yoon Han, Joonhong Park

**Affiliations:** 1 Department of Pediatrics, College of Medicine, The Catholic University of Korea, Seoul 06591, Korea; han024@catholic.ac.kr; 2 Department of Laboratory Medicine, Jeonbuk National University Medical School and Hospital, Jeonju 54907, Korea; 3 Research Institute of Clinical Medicine of Jeonbuk National University-Biomedical Research Institute of Jeonbuk National University Hospital, Jeonju 54907, Korea

**Keywords:** terminal 14q32 duplication, developmental delay, intellectual disability, growth retardation, facial dysmorphism, cerebral infarction, exome sequencing, chromosomal microarray

## Abstract

The terminal 14q32 duplication has been reported often in association with other cytogenetic abnormalities, and individuals with this specific duplication showed varying degrees of developmental delay/intellectual disability (DD/ID) and growth retardation (GR), and distinct facial dysmorphisms. Herein, based on the limited cases of terminal duplication of 14q32 known to date, we present new affected siblings presenting with DD/ID, GR, and facial dysmorphism, as well as cerebral infarction caused by recurrent de novo der(14)t(14;14)(p11.2;q32.1) leading to terminal duplication of 14q32. We used coverage analysis generated via duo exome sequencing, performed chromosomal microarray (CMA) as a confirmatory test, and compared our findings with those reported previously. Coverage analysis generated via duo exome sequencing revealed a 17.2 Mb heterozygous duplication at chromosome 14q32.11-q32.33 with a Z ratio ranging between 0.5 and 1 in the proband and her elder brother. As a complementary method, CMA established a terminal duplication described as the arr[hg19]14q32.11q32.33(90,043,558_107,258,824)x3 in the proband and her elder brother; however, the parents and other siblings showed normal karyotyping and no abnormal gain or loss of CMA results. Five candidate genes, *BCL11B*, *CCNK*, *YY1*, *DYNC1H1*, and *PACS2*, were associated with the clinical phenotypes in our cases. Although the parents had normal chromosomes, two affected cases carrying terminal duplication of 14q32 can be explained by gonadal mosaicism. Further studies are needed to establish the association between cerebrovascular events and terminal duplication of chromosome 14q32, including investigation into the cytogenetics of patients with precise clinical descriptions.

## 1. Introduction

Chromosome 14 is an acrocentric chromosome. Its short arm consists of only of the satellite that is extremely gene-poor and involved in various chromosomal rearrangement, including translocation. The finished sequence of human chromosome 14 comprises 87,410,661 base pairs, representing 100% of its euchromatic portion, in a single continuous segment covering the entire long arm with no gaps. The segment contains between 800 and 1300 genes and constitutes nearly 3.5 percent of the total cellular DNA [1]. On the other hand, chromosomal abnormalities are a significant cause of human genetic disorders and are associated with various complex traits [2]. Particularly, the karyotype imbalance occurs in cases of numerical anomalies, such as missing one (or more) chromosome(s) (monosomy) or excess (trisomy). Otherwise, disproportionate structural changes occur due to deletion or duplication of chromosome segment(s). In an unbalanced chromosomal aberration, the chromosomal complement carries an incorrect number of chromosomal components, leading to serious and multiple congenital anomalies [3]. Duplication is one of the structural alterations underlying the imbalance. Duplications involve repetition of a segment of chromosome once or several times, depending on whether the duplicated segments lie in the same orientation (tandem) or become flipped (inverted). Duplications can lead to an increased copy number of genes and an associated increase in their expression [4].

To date, the various segments of abnormal rearrangements in 14q have been reported [5,6,7,8,9,10,11,12,13,14,15,16,17,18,19,20,21,22,23,24,25,26,27,28,29,30,31,32,33,34,35,36,37,38,39,40,41,42,43,44,45,46,47,48,49], and the terminal 14q32 duplication has been reported often in association with other cytogenetic abnormalities [7,8,11,43,47]. The genotype/phenotype correlations remain unclear, despite success in previous studies [39,42,43,47,49]. Individuals with this specific duplication showed varying degrees of developmental delay/intellectual disability (DD/ID) and growth retardation (GR), and distinct facial dysmorphisms [39,42,43,47,49]. Herein, based on the limited cases of terminal duplication of 14q32 known to date, we present new affected siblings presenting with DD/ID, GR, and facial dysmorphism, as well as cerebral infarction (CI) caused by recurrent de novo der(14)t(14;14)(p11.2;q32.1) leading to terminal duplication of 14q32. We used coverage analysis generated via duo exome sequencing, performed chromosomal microarray (CMA) as a confirmatory test, and compared our findings with those reported previously.

## 2. Materials and Methods

### 2.1. Duo Exome Sequencing of Two Affected Siblings

To identify the possible genetic causes of CI, growth retardation, and/or ID in patients, the exomic DNA of two affected siblings were amplified using Agilent’s SureSelect XT Human All Exon v5 (Agilent Technologies, Santa Clara, CA, USA). Based on suspected neurodevelopmental diseases, paired-end sequencing was performed to identify the genetic alterations using the Illumina NextSeq 550 System (Illumina, San Diego, CA, USA) at the Green Cross Genome (Yongin, Korea). Base calling, quality check, read alignment, variant calling, and variant annotation for functional interpretation of results were carried out according to GATK Best Practices workflow for germline short variant discovery (https://gatk.broadinstitute.org/hc/en-us; accessed on 22 August 2020). Interpretation of sequence variants was reviewed manually by medical laboratory geneticists based on the Joint Consensus Recommendation of the American College of Medical Genetics and Genomics and the Association for Molecular Pathology standards and guidelines [50]. The inclusion criteria for candidate variants expected to be associated with clinical manifestations were: read depth > 30×, Phred quality score > 20, absence of Fisher strand bias, allele frequency < 0.01%, indel or nonsynonymous base changes occurring at the exon–intron boundaries or coding region, dominant heterozygous, compound heterozygous, or homozygous recessive status in both siblings, and previously associated with DD/ID, GR, facial dysmorphism, and/or CI in the Clinvar (https://www.ncbi.nlm.nih.gov/clinvar/; accessed on 2 April 2021) or Online Mendelian Inheritance in Man (OMIM, https://www.omim.org/; accessed on 2 April 2021) databases. All candidate variants were confirmed visually using an Integrative Genomics Viewer followed by Sanger sequencing with ABI3500XL Genetic Analyzer (Applied Biosystems, Foster City, CA, USA). In addition, copy number variations (CNVs) are a source of human genetic alteration and have been reported previously as a genetic cause of unexplained DD/ID, GR, facial dysmorphism, and/or CI [51,52,53]. VisCap, inference and visualization tool of germline CNVs from targeted clinical sequencing data, were used for additional depth-of-coverage analysis of exome sequencing data [54].

### 2.2. Chromosomal Microarray

To validate the CNV detected by depth-of-coverage analysis using exome sequencing, a whole genomic screening of chromosomal rearrangements by CMA was performed using SurePrint G3 Human CGH + SNP Microarray 4×180K (Agilent Technologies, Santa Clara, CA, USA), according to the manufacturers’ protocol. All the samples were matched with Human Genomic DNA reference (Agilent Technologies or Promega, Madison, WI, USA). Data were acquired using the Agilent Feature Extraction software 12.0.2.2 and Agilent CytoGenomics 4.0 and inspected visually using the Agilent Genomic Workbench Software 7.0.4.0 and Agilent CytoGenomics 4.0. Genomic locations were mapped using the human genomic reference sequence GRCh37/hg19. CNVs were classified using the ADM-2 algorithm with a minimum size filter cut-off of 200 Kb for copy number gains or losses in the region, and a minimal absolute average log2 ratio of 0.25 as the cut-off, copy number gain (0.25), or loss (−0.25) for heterozygous regions.

## 3. Case Presentation

**Figure 1:**
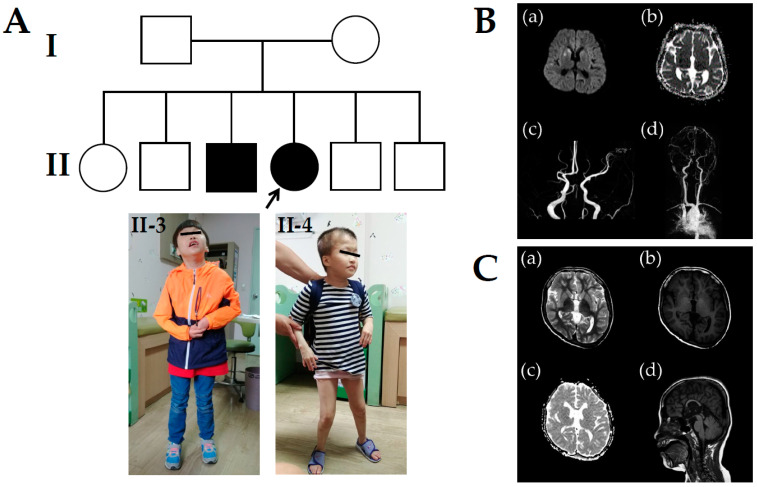
Pedigree analysis, clinical imaging, and brain magnetic resonance imaging (MRI) and angiography (MRA) in the proband (II-4) and her elder brother (II-3). (**A**) Pedigree analysis and clinical photography. (Upper panel) Family pedigree shows a recurrent and dominant de novo duplication of 14q32.11-q32.33 in Korean siblings associated with intellectual disability, growth retardation, facial dysmorphism, and cerebral infarction. (Lower panel) A dysmorphic face characterized by frontal bossing, widely spaced teeth, broad upper alveolar ridges, broad mouth, downturned corners of the mouth, broad nasal root, and deep nasal bridge. (**B**) The proband showed acute infarction in the right striatocapsular region and cortical area of the right temporal lobe (**a** and **b**). The proximal M1 segment of right MCA was occluded, and the distal MCA was retrograde filled by leptomeningeal collaterals (**c** and **d**). (**C**) Her elder brother showed abnormal development of the right hemisphere combined with cortical dysplasia (**a** and **b**) and dysgenesis of the corpus callosum (**c** and **d**).

**Figure 2:**
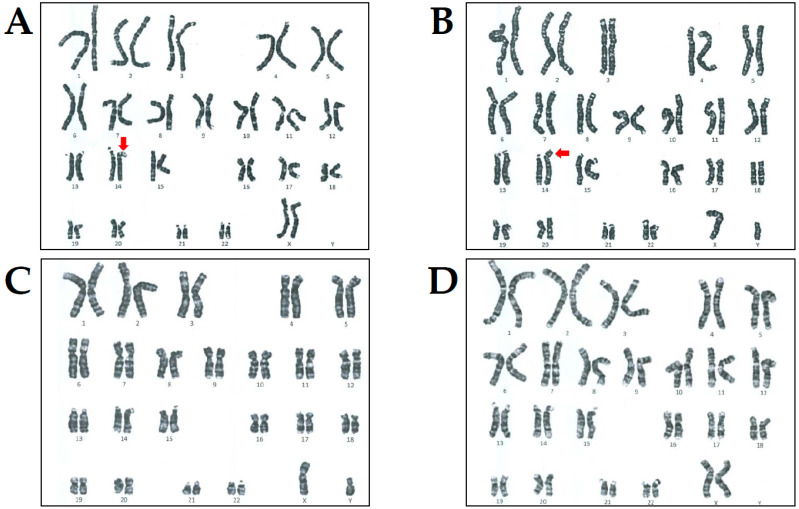
Results of chromosomal analysis in the proband and her family members. Chromosomal analysis revealed 46,XX,der(14)t(14;14)(p11.2;q32.1) in the proband (**A**) and 46,XY,der(14)t(14;14)(p11.2;q32.1) in her elder brother (**B**) as indicated by the red arrow, respectively. However, their father (**C**) and mother (**D**) showed normal karyotyping.

The 14-year-old female proband (II-4 in Figure 1A) presented with acute left arm weakness and was referred to the Department of Pediatric Neurology, Daejeon St. Mary’s Hospital (Daejeon, Korea). On admittance, she was diagnosed with severe DD/ID with an intelligence quotient (IQ) of 40 and GR similar to her elder brother (II-3 in Figure 1A). She was born uneventfully to healthy, unrelated Korean parents after a normal pregnancy. Birth weight was 2750 g (20th percentile) and head circumference was 33 cm (25th percentile). Prenatal history was unremarkable. The patient’s height was 99 cm (<3 percentile), weight was 14 kg (<3 percentile), and head circumference was 51 cm (<3 percentile) at 14 years of age. Her early development was markedly delayed. She could not utter a single word and used some onomatopoeic words. She performed head control at 6 months and walked independently at 30 months. She showed a dysmorphic face, including frontal bossing, widely spaced teeth, broad upper alveolar ridges, broad mouth, downturned corners of the mouth, broad nasal root, and deep nasal bridge. She carried sparse hair, eyelashes, and eyebrows. She did not develop secondary sexual characteristics, such as breast enlargements or pubic hair, until the age of 18. Levels of growth and sex hormones were low, whereas other pituitary hormones were within normal range. On physical examination, cardiac auscultation revealed high-pitched sounds, and an early diastolic decrescendo murmur heard best at the third intercostal space on the left. She had atopic dermatitis of the inside crease of the elbow, popliteal areas, face, and neck. Brain magnetic resonance imaging (MRI) revealed acute infarction in the right striatocapsular region and cortical areas of right temporal lobe (**a** and **b** in Figure 1B). MR angiography showed that the M1 segment of the middle cerebral artery (MCA) was occluded and distal MCA retrograde filled by leptomeningeal collaterals (**c** in Figure 1B). Cervical spine radiography indicated no cervical subluxation or cervical abnormality (**d** in Figure 1B). Thoracic and transesophageal echocardiogram revealed severe aortic regurgitation (AR) with mild aortic stenosis (AS) and dilated ascending aorta. Thrombotic profiles including homocysteine, antithrombin III, protein C and S activities, activated protein C resistance, factor V Leiden, Factors II, VII, VIII, and X, and plasminogen activity were within the normal range. Immunological tests including lupus anticoagulant, anticardiolipin antibodies, antinuclear antibodies, antiphospholipid antibodies, anti-SS-A/B antibodies, and ANCA were negative. Routine laboratory and radiology tests were within normal limits and other metabolic parameters, including amino acid levels, organic acids, lactate/pyruvate, thyroid hormones, and α-galactosidase were within normal ranges. Fragile X testing was negative. However, chromosomal analysis revealed de novo 46,XX,der(14)t(14;14)(p11.2;q32.1) according to the guidelines of the International System for Human Cytogenomic Nomenclature (ISCN, 2016) (Figure 2A). The patient was managed conservatively with intravenous fluids, mannitol, and dexamethasone and started on aspirin (100 mg daily) for secondary stroke prevention. Weakness gradually improved, and the patient regained normal health status after 1 month. She was administered enalapril for AR treatment.

The proband’s elder brother (II-3 in Figure 1A) visited the Department of Pediatric Neurology, Daejeon St. Mary’s Hospital for genetic counseling and segregation analysis at the age of 19 years. On admittance, he was also diagnosed with severe DD/ID, IQ of 50, and GR. He was born via normal spontaneous delivery at 38 + 1 weeks of gestation. His birth weight was 2800 g (25th percentile), and his head circumference was 33 cm (25th percentile). He manifested left hemiparesis after birth and persistent left arm weakness. He could control his head well at the age of 7 months and roll over at the age of 11 months. He sat independently at 15 months, crawled at 19 months, and stood at 23 months. Walking was accomplished at the age of 30 months. Speech was limited to pronouncing just his names at the age of 4. His weight at 19 years of age was 25 kg (<third percentile) and height 119 cm (<third percentile). The face showed slightly coarse features with widely spaced teeth, broad mouth, downturned corners of the mouth, broad nasal root, and deep nasal bridge. He could only speak a few words. He did not show any secondary sexual characteristics, including pubic hair, deepening of voice, or increased muscle mass and strength, at the age of 20. The levels of growth and sex hormones were low, whereas other pituitary hormones were within the normal range. Brain MRI showed dysgenesis of the corpus callosum, and the right hemisphere was abnormally smaller than the other (**a**–**d** in Figure 1C). Laboratory tests such as thyroid function and metabolic profiles, and radiologic findings were normal. Fragile X testing was negative. However, chromosomal analysis revealed de novo 46,XY,der(14)t(14;14)(p11.2;q32.1) similar to the proband according to the guidelines of the ISCN (Figure 2B).

## 4. Results

**Figure 3:**
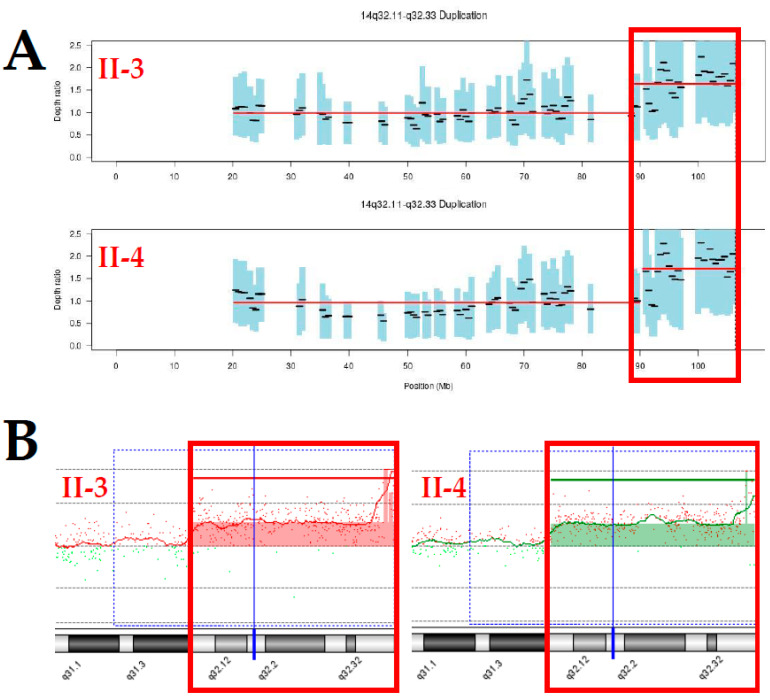
Results of coverage analysis based on duo exome sequencing, and chromosomal microarray in the proband (II-4) and her elder brother (II-3). (**A**) Copy number analysis using duo exome sequencing revealed 14q32.11-q32.33 duplication in the proband (II-4) and her elder brother (II-3), as highlighted in the red box. (**B**) Chromosomal microarray established a gain of 17.2 Mb from 14q32.11 to 14qter in the proband (II-4) and her elder brother (II-3). The arr[hg19]14q32.11q32.33(90,043,558_107,258,824)x3 is highlighted in the red box.

On-target yields of 3,293,954,491 and 3,127,800,131 reads were generated from the proband and her elder brother based on the quality of all sequences. The mean read depths (×) were 65 and 62, and the percentage of bases above 30× was 81% and 85%, respectively. As a result, heterozygous variants of the two candidate genes associated with DD/ID, GR, or facial dysmorphism were identified via duo exome sequencing in both the proband and her elder brother: *FRMD4A* (NM_018027.4: c.2296G>A/p.Ala766Thr) located on chr10:13,699,293 related to corpus callosum, agenesis, with facial anomalies, cerebellar ataxia (OMIM ID: #616819), and *TBX1* (NM_080647.1: c.1480C>G/p.Pro494Ala) located on chr22:19,754,382 related to conotruncal anomaly face syndrome (OMIM ID: #217095). On the other hand, coverage analysis generated via duo exome sequencing revealed a 17.2 Mb heterozygous duplication at chromosome 14q32.11-q32.33 with a Z ratio ranging between 0.5 and 1 in the proband and her elder brother (Figure 3A). As a complementary method, CMA established a terminal duplication from 14q32.11 to 14qter described as the arr[hg19]14q32.11q32.33(90,043,558_107,258,824)x3 in the proband and her elder brother, similar to the de novo der(14)t(14;14)(p11.2;q32.1) by chromosomal analysis (Figure 3B). To investigate its genetic origin in recurrent der(14)t(14;14)(p11.2;q32.1), genetic counseling and segregation analysis of parents and other siblings of the proband was performed. However the parents and other siblings showed normal karyotyping and no abnormal gain or loss of CMA results. CMA ratio plots for chromosome 14 revealed no imbalance in both parents of the proband. In addition, heterozygous variants of the two protein-coding genes located on chromosome 14q32.11-q32.33 were identified by duo exome sequencing in both the proband and her elder brother: *BEGAIN* (NM_020836.3:c.1195A>G/p.Ser399Gly; rs753726540) located on chr14:101,004,893 and *AHNAK2* (NM_138420.2:c.10856G>A/p.Arg3619Gln; rs752295555) located on chr14:105,410,932.

## 5. Discussion

**Figure 4:**
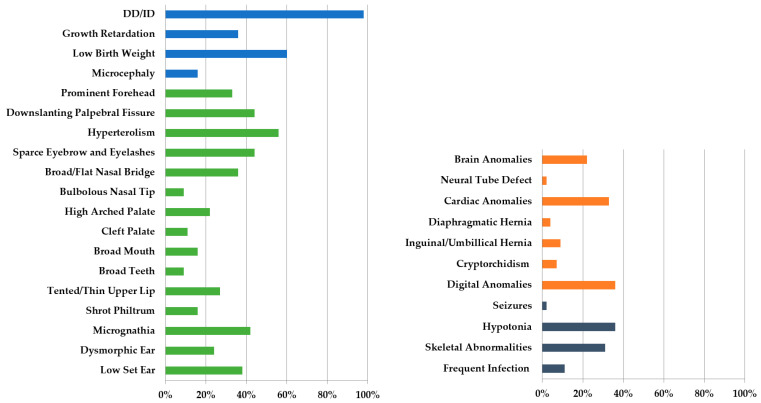
Literature review of the clinical features and frequencies of 14q duplication in 45 reported cases attributed to translocation, tandem duplication, inversion, or insertion [5,6,7,8,9,10,11,12,13,14,15,16,17,18,19,20,21,22,23,24,25,26,27,28,29,30,31,32,33,34,35,36,37,38,39,40,41,42,43,44,45,46,47,48,49]. Blue bar, detailed phenotypes of growth and development; green bar, detailed phenotypes of facial dysmorphisms; orange bar, detailed phenotypes of other organ system anomalies; grey bar, detailed phenotypes of other problems; DD, developmental delay; ID, intellectual disability.

Chromosome 14, one of five acrocentric chromosomes in the human genome, contains a heterochromatic short arm carrying ribosomal RNA genes essentially, and a euchromatic long arm in which most of the protein-coding genes are located. Phenotypic heterogeneity has been observed in children with partial duplication of various chromosomal segments. Whenever a larger or more distal segment of the long arm was involved, clinical features tended to be more severe. However, it is not clear whether substantial phenotypic variations between patients were due in part to individual differences, environmental factors, and the size of the trisomic segment, or the associated partial trisomy [55,56]. Duplication of the distal segment was not strongly linked to a well-described syndrome. Particularly, the chromosomal abnormality affecting the proximal segment of 14q has been commonly reported, while terminal 14q32 duplication is rarely associated with the monosomic segment of other chromosomes. Since Allerdice et al. first reported a case of partial trisomy of 14q [5], a few live-born patients have been published. Distal trisomy 14 is rarer than proximal trisomy 14, and full trisomy 14 most often causes stillbirth. Several cases involve DECIPHER’s genome database, with a brief phenotypic description (https://decipher.sanger.ac.uk/; accessed on 2 April 2021), and approximately 20 cases of pure 14q duplication are reported in the literature [14,28,31,34,39,40,42,44,45,47,48,49]. Despite the rarity of terminal 14q32 duplication, a distinct phenotype characterized by low birth weight, GR, DD/ID, hypotonia, and facial dysmorphisms has emerged. A systematic literature review of clinical features and frequencies in 45 reported cases with 14q duplication was conducted [5,6,7,8,9,10,11,12,13,14,15,16,17,18,19,20,21,22,23,24,25,26,27,28,29,30,31,32,33,34,35,36,37,38,39,40,41,42,43,44,45,46,47,48,49] (Supplementary Table S1). The unique phenotype was characterized by DD/ID (98%), low birth weight (60%), GR (36%) (blue bars in Figure 4), and facial dysmorphisms, including downslanting palpebral fissure, hyperteolorism, broad and/or flat nasal bridge, micrognathia, low-set ear, and sparse eyebrows and eyelashes (green bars in Figure 4). The frequently accompanying clinical manifestations include brain anomalies (22%), cardiac anomalies (33%), digital anomalies (36%) involving organ systems (orange bars in Figure 4), hypotonia (36%), and skeletal abnormalities (31%), among other conditions (grey bars in Figure 4).

On the other hand, duplication of 14q occurs as a result of various chromosomal structural aberrations, such as translocation, tandem duplication, inversion, or insertion [5,6,7,8,9,10,11,12,13,14,15,16,17,18,19,20,21,22,23,24,25,26,27,28,29,30,31,32,33,34,35,36,37,38,39,40,41,42,43,44,45,46,47,48,49]. In most reported cases (69%), the abnormal chromosome segment resulted from a carrier parent. In 24 cases (57%), the 14q duplication is attributed to parental translocation (83%) and is associated with partial monosomy. The origin translocation was paternal in 17 cases (50%) and maternal in 17 cases (50%). De novo translocation was observed in five cases (17%), including our patients. Balanced reciprocal translocations and their unbalanced offspring have been reported for the first time [6]. In most cases, the unbalanced karyotype was the result of genetic duplication or translocation deficiency of the chromosomes derived from the parents. Pericentric inversion occurred in about 10% of patients. Pericentric inversions of chromosome 14 are very uncommon events, and only four such cases were reported with recombinant progeny [38,45,57]. Unbalanced chromosomal recombination results from crossing over within a pericentric inversion in acrocentric chromosomes under specific conditions. However, duplications and deficiencies of the acrocentric short arm have little or no phenotype impact. Congenital anomalies in an individual with a recombined inverted acrocentric chromosome can be attributed specifically to either partial duplication or deletion of the specific chromosomal long arm. It is interesting to find that breakpoints, size of duplicated segment, and the involved genes vary among patients with this chromosomal rearrangement. Eight cases (10%) reported de novo tandem duplication. Intrachromosomal or extrachromosomal duplications are very frequent in the human genome; however, intrachromosomal duplications of the same chromosomal segment (tandem duplications), which can be serial (direct) or inverted (mirror), are infrequent in chromosome 14q [15,21]. Only one case of insertion (2%) was reported [29]. The breakpoints are variable, and the genetic heterogeneity may explain the lack of a well-defined clinical syndrome [32].

The clinical features of the present case were compared with those described in the literature to identify a minimal overlapping region characterized from a molecular genetics perspective, including disease-associated genes. First, heterozygous *FRMD4A* and *TBX1* variants on chromosomes 10 and 22, respectively, identified in both affected siblings were excluded as a cause of clinical manifestations because these genes act in an autosomal recessive manner. Second, sequence mutations in some genes located on 14q have been reported in disease syndromes (such as Krabbe disease, spinocerebellar ataxia with axonal neuropathy, and Leber congenital amaurosis) [45], but the phenotype of our cases does not match with the studies reported previously. Similarly, two protein-coding genes, *BEGAIN* and *AHNAK2,* located on this region have yet to be linked to clinical manifestations in both affected cases, and these two genes and their associated clinical symptoms were not described in OMIM or elsewhere. However, the distal region of chromosome 14q32 duplicated in our cases involves nearly 120 genes according to Ensembl Genome Browser, and duplicated segments between 14q32and 14q33 were frequently reported by DECIPHER’s genome database and ClinVar. As a result, several protein-coding genes associated with DD identified by DECIPHER’s genome browser were expected to be related to DD/ID, GR, and facial dysmorphism (https://decipher.sanger.ac.uk/browser#q/12:66711-3409277/location/12:1-7952122; accessed on 2 April 2021) [58]. Five out of 15 candidate genes were associated with the clinical phenotypes in our cases (Table 1).

**Figure 5:**
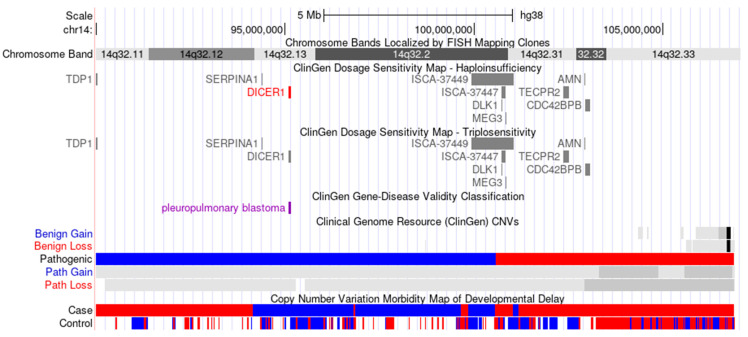
Schematic representation of previously reported cases spanning the terminal 17.2 Mb of chromosome 14q32, including ClinGen Dosage Sensitivity Map and Copy Number variation Morbidity Map of Developmental Delay displayed by UCSC Genome Browser (http://genome.ucsc.edu/; accessed on 30 August 2021). Blue bars indicate pathogenic or related gains based on copy number variation morbidity map of developmental delay.

Particularly, heterozygous mutations in the *BCL11B* gene located on chr14:99,169,286-99,272,196 (OMIM #618092) can induce DD/ID with speech delay, dysmorphic facies, and T-cell abnormalities [59,60]. Heterozygous mutations in the *CCNK* gene located on chr14:99,481,408-99,512,439 (OMIM #618147) can lead to DD/ID with hypertelorism and distinctive facies [61]. Heterozygous mutation involving the *YY1* gene located on chr14:100,239,143-100,282,787 (OMIM #617557) can cause Gabriele-de Vries syndrome, characterized by mild-to-profound DD/ID in all affected individuals and a broad spectrum of morphological and functional abnormalities [62]. Heterozygous mutations in the *DYNC1H1* gene located on chr14:101,964,572-102,056,442 (OMIM #614563) can lead to autosomal dominant mental retardation-13 with cerebral cortical malformations or microcephaly [63,64]. Heterozygous mutations involving the *PACS2* gene located on chr14:105,300,717-105,398,146 (OMIM #618067) cause developmental and epileptic encephalopathy-66 with facial dysmorphism and cerebellar dysgenesis, characterized by the onset of various types of seizures in the first days or weeks of life [65]. However, these curated genes/regions are not predicted to be dosage-sensitive according to ClinGen Dosage Sensitivity Map: ClinGen Triplosensitivity Score for the *TECPR2* gene is 0 and 14 other genes are awaiting review (https://dosage.clinicalgenome.org/; accessed on 30 August 2021) (Figure 5).

On the other hand, imprinted regions were identified at 14q32 in the duplicated segment. The imprinted genes may be maternal (*MEG3* and *MEG8*) or paternal inherited (*DLK1* and *RTL1*) [66]. Consistent with these findings, both paternal and maternal uniparental disomies for chromosome 14 cause distinct clinical features. However, our cases were not affected by uniparental disomy. No substantial differences in the clinical manifestations were found, underlining the lack of imprinted gene contribution in the phenotypic features [39]. Furthermore, the gene-regulatory interactions and the epigenetic mechanisms are not understood completely, and their role in the clinical symptoms remains unknown.

## 6. Conclusions

In the present study, the proband with DD/ID, GR, and facial dysmorphism showed CI, and her elder brother carried a history of suspected perinatal CI, in contrast to previous reports. Although the parents had normal chromosomes, two affected cases carrying terminal duplication of 14q32 can be explained by gonadal mosaicism. The proportion of abnormal germ cells is determined by the mutations early in germ cell differentiation. Further studies are needed to establish the association between cerebrovascular events and terminal duplication of chromosome 14q32, including investigation into the cytogenetics of patients with precise clinical descriptions.

## Figures and Tables

**Table 1:** Fifteen candidate genes associated with developmental delay located between 14q32 and 14q33.

DD Genes	Morbidity	Diseases	OMIM	Inheritance
*CCDC88C*	Yes	Hydrocephalus, congenital, 1	# 236600	AR
*TRIP11*	Yes	Achondrogenesis, type IA Odontochondrodysplasia 1	# 200600 # 184260	AR
*SLC24A4*	Yes	Amelogenesis imperfecta, type IIA5	# 615887	AR
*TMEM251*	Yes	Dysostosis multiplex, Ain-Naz type	# 619345	AR
*UBR7*	Yes	Li-Campeau syndrome	# 619189	AR
*VRK1*	Yes	Pontocerebellar hypoplasia type 1A	# 607596	AR
*BCL11B*	Yes	Intellectual developmental disorder with dysmorphic facies, speech delay, and T-cell abnormalities	# 618092	AD
*CCNK*	Yes	Intellectual developmental disorder with hypertelorism and distinctive facies	# 618147	AD
*YY1*	Yes	Gabriele-de Vries syndrome	# 617557	AD
*DYNC1H1*	Yes	Mental retardation, autosomal dominant 13	# 614563	AD
*TECPR2*	Yes	Spastic paraplegia 49, autosomal recessive	# 615031	AR
*APOPT1*	Yes	Mitochondrial complex IV deficiency, nuclear type 17	# 619061	AR
*AKT1*	Yes	Cowden syndrome 6	# 615109	AD
*BRF1*	Yes	Cerebellofaciodental syndrome	# 616202	AR
*PACS2*	Yes	Developmental and epileptic encephalopathy 66	# 618067	AD

DD, developmental delay; OMIM, Online Mendelian Inheritance in Man; #, a number sign in OMIM; AR, autosomal recessive; AD, autosomal dominant.

## Data Availability

The data presented in this study are available on request from the corresponding author.

## Supplementary Materials

The following are available online at https://www.mdpi.com/article/10.3390/genes12091388/s1, Table S1: Detailed rearranged region of chromosome 14q and clinical magnifications from literature review in patients with 14q duplication.
