# Supplementary material for: A Recurrent De Novo Terminal Duplication of 14q32 in Korean Siblings Associated with Developmental Delay and Intellectual Disability, Growth Retardation, Facial Dysmorphism, and Cerebral Infarction: A Case Report and Literature Review
Source: Genes (Basel). 2021 Sep 7;12(9):1388. doi: 10.3390/genes12091388 (PMC8472681; doi:10.3390/genes12091388)
Supplement: Supplementary file 1 [file genes-12-01388-s001.zip › genes-1351370-supplementary.pdf]

Supplementary Table S1. Detailed rearranged region of chromosome 14q and clinical magnifications from literature review in patients with 14q duplication

Literatures	Rearranged region	Sex	Age	Clinical magnifications
Translocation				
**Allderdice (1971)**	t(6q,20p?)+,t(14q,6q)+mat	F	4y	eye-small, deep-set, close, ptosis, low-set ears, small chin, narrow arched palate high-pitched cry, and electrocardiogrphic abnormality
**Short (1972)**	t(9p+;14q-)-mat	M	3d	microcephaly, small fontanelle, sloping forehead<br>low set and dysmorphic ear, nasal beaking, hypoplastic mandible, short neck, barrel chest, kyphotic spine, cryptotorchidism, equinovarus deformity, valgus deformity, and cardiomegaly
**Reiss (1972)**	t(2q+;14q-)-pat	M	10m	low birth weight, dolicocephaly, small palpebral fissures, Exotropia, retrocessed ears, increased nasolabial distance, upturned nares, cleft soft palate, large mouth, micrognathia, small finger nail, cryptorchidism, inguinal hernia, and generalized hypertonia and hyperreflexia
**Pfeiffer (1973)**	t(14q-;21q+)-mat	F	15m	hypotonia, brachycephaly, antimogoloid slant, congenital glaucoma, and severe developmental delay
**Fryns (1974)**	t(14q-,19+)-pat	F	7y	low birth weight, growth retardation, intellectual disability, hyperterolism, broad base of nose, fish-mouth, high arched palate, low set-hairline in the neck, truncal obesity, and fusiform legs and fingers
**Wahlstrom (1974)**	t(14;22)(q22 or 23;q13)-pat	NA	NA	malformations of the arthrogryposis type, and heart enlargement
**Turleau (1975)**	t(12;14)(q24.4;q21)-mat	M	18m	oval face, distinct form of mouth, prominent nose, psychomotor and growth retardation, micropthalmia, cardiopathy, anomaly of extrimities, ptosis, microcephaly, cleft palate, and club foot
**Raoul (1975)**	t(10;14)(q15.2;q22)-mat	F	18m	growth retardation, intellectual disability, prominent mouth, cleft palate, and club foot
**Simpson (1977)**	t(3;14)(q29;q21)-mat	F	14m	global developmental delay, epicanthal folds, antimongoloid slant, ptosis, hyperterolism, asymmetry of head, low-set ear, triangular shaped mouth, high and narrow palate, and bifid uvula
**Fryns (1977)**	t(14q-,11q+)-pat	M	2.5m	low birth weight, hyperterolism, bilateral epicanthus, anteroverted nostrils, low set ears, micrognathia, neck-retroflexion, bilateral cryptorchidism, small penis with short preputium, and malposition of the fingers of both hands

**Birdgman (1980)**	t(10;14)(p14;q22) *de novo*	F	5m	low birth weight, low set ear, wide nasal bridge, a narrow palpebral fissure, ptosis of the left lid, prominent upper lip, widely spaced nipples, and tetralogy of Fallot
**Geomaneanu (1981)**	t(5;14)(q13;q23q32)-mat	M	2m	facial dysmorhism, truncus arteriosus, and agenesis of corpus callosum
**Sklower (1982)**	t(14;18)(q31.1;q32)-mat	M	22y	high nasal bridge, beaked nasal tip, extremely short philtrum, arched palate, clino-camptodactyly, bilateral pilonidal dimples, microcephaly, broad high forehead, hyperterolism, deep orbits and exophtalmos, sparse eyebrows and eyelashes, thin upper lip, widely spaced teeth, occipital hair whorl, lumbar scoliosis, gracile extrimities, joint stiffness, radial-ulnar synostosis, tapered digits, and severe myopia
**Cohen (1983)**	t(14;X)-mat	F	8m	low birth weight, frontal bossing, large anterior and posterior fontanelle, triangular-shaped face<br>shallow orbit, "carp-shaped" mouth, mild micrognathia, low set ear, nipple-hypoplastic and laterally displaced syndactyly (2nd 3rd toes), and dimple at shoulder and elbow
**Romain (1983)**	t(11;14)(q25;q24)-mat	M	1m	low birth weight, hypotonia, prominent occiput, narrow bifrontal areas, large anterior and posterior fontanelle, dysplastic ear, micrognathia, carp-shaped mouth, narrow palatal arch, and broad nose
**Atkin (1983)**	t(14;15)(q24.3;26.1)-pat	M	8m	growth failure, prominent occiput, enlarged ant. Fontanelle, hyperterolism, downward slant of palpebral fissures, posteriorly angulated ears, flat nasal bridge, "cupid bow" upper lip, high arched palate, micrognathia, hypotonia, hyporeflexia, inward deviation of toes, developmental delay, microconea, severe myopia, and bilateral exotropia
**Turleau (1983)**	t(10;14)(q26;q32.1)-pat	M	22m	intrauterine growth retardation, growth retardation, craniofacial dysmorphism, large face, chubby cheeks, facial asymmetry, hyperterolism, broad nose, short prominent philtrum, carp-shaped mouth, micrognathia, low set ear with a promient antitagus, abnormal position of nipples, and malformations-brain, lung and heart
**Sklower (1984)**	t(14;18)(q31;q23)-mat	M	22y	low birth weight, sparse eyelashes and eyebrow, macular eruption, developmental delay, non-insulin dependent diabetes mellitus, growth retardation, frontal bossing, hyperterolism, exophthalmos, short philtrum, thin cupid bow lip, micrognathia, postortated ears, radioulnar synostosis, metacarpal phalangeal joint limitation. broad sacrum with pilonidal dimples, scoliosis, valgus deformity, myopia, dysarthria, sensory deafness, hypotonia, hyporeflexia, poor fine and gross motor coordination, poor visual motor coordination, and frequent infection

	t(14;18)(q24;q11)-mat	M	5m	antimongoloid slant, hyperterolism, epicanthal fold, short philtrum, thin cupid bow upper lip, micrognathia, broad nasal bridge, low set year, developmental delay, hypotonia, hearing impairment, electrocardiographic abnormality, and frequent infection
**Markkanen (1984)**	t(7;14)(q36;q24)-mat	M	3m	low birth weight, abnormal shape of skull, enlarged fontanelle, broad sutures, hyperterolism, mongoloid slant, coloboma, prominent nose, narrow palate, hypertrophied gums, micrognathia, small and low set ear, bilateral coxa luxation, contractures of several joints, club foot, broad hands, clinodactyly of the 5th finger, brain atrophy, and dilatation of right atrium and hypertrophy of ventricle
**Kaiser (1984)**	t(7;14)(q36;q24)-mat	M	10m	low birth weight, hyperterolism, lateralization of canthus, antimongoloid palpebral fissures, hypoplastic lower eyelid, brush-field spots on iris, retroverted low set ear, hypoplastic helices and absent lobuli, nose-short high bridge, prominent columella and anteverted nostrils, micrognathia, hypotonia, and scoliosis
**Wakita (1988)**	t(5;14)(p15.33;q31.2)- mat	M	1m	preterm birth, facial asymmetry, frontal bossing, hyperterolism, cloud left cornea, cupid bow upper lip, micrognathia, low set ear-prominent antihelices and atresia of both external auditory canal, pigeon chest, overlapping finger, clinodactyly of the 5th fingers, prominent calcaneus, and hypotonia
**Gilgenkrant (1990)**	t(14;14)(qter→q24:p13→qter) *de novo*	F	1m	low birth weight, large fontanelle with wide sutures and ossification, nose-thin, parrot-like with anteroverted nostrils, micrognathia, hypoplasia of zygomatic arch, short philtrum with a tended, prominent upper lip, sparse hair, asymmetric and downslanting palpebral fissures, hypoplastic eyelids, lower lid defects, colobomata of the lt. iris, bilateral slight micropthalmia, lt. embryotoxon, corneal dystrophy, rt. cataract, keratitis, bilateral clinodactyly of the 5th finger, large toes asymmetric nipples, sacroccoccygeal pit, cardiac anomaly, hypotonia, and intellectual disability
**Nakamira (1990)**	t(14;17)(17pter 17q25.3::14q24.3-14qter) *de novo*	M	1m	low birth weight, protruding occiput, deformed ears, large face, broad nose, thin eyebrow, Mongoloid slant, fistula-preauricular region, protruding maxilla, wide sagittal suture, hypertelorism, high arched palate, abnormal teeth, hypospadias, right ventricle hypertrophy, small ductus arteriosus, poor weight gain, and fingerprint abnormality
**Sonada (2001)**	rec(14),dup(14q), der(14)(14pter→cen:14q32.1	M	4	low birth weight, prominent forehead, hypertelorism, downward slanted palabral fissures, strabismus, macrostomia, downturning corners of the mouth, high nasal

	1→14p12:14q32.11→14qter) - mat			root, micrognathia, low set and port angulated ears, cleft palate, cryptorchidism, clinidactyly (5th fingers), overlapping fingers, dysplastic optic nerves, hypotonia, growth retardation, developmental delay
**Orellana (2001)**	t(X;14) *de novo*	F	18m	low birth weight, hypotonia, low set and dysplastic ears, retrognathia, macrognathia, protruding upper lip, bilateral corneal leucoma, coloboma of the iris, short arm, small hands and feet, simian creases, antimongoloid features, syndactyly (2nd and 3rd toes), and clinodactyly (5th fingers)
**Sutton (2002)**	t(14;22)(q32.3;p11.2)- mat	F	3y	low birth weight, lumbosacral myelomeningocele, partial agenesis of the corpus callosum, cardiac abnormalities, hyperterolism, tented mouth, simple ears, small mandible, hypotonia, and growth retardation
**Perrin (2002)**	t(3;14)(p25;q24)-pat	F	15m	enlargement of anterior fontanelle, hyperterolism, broad nasal bridge, high arched palate, micrognathia, low set ears, short neck, overriding fingers, rocker bottom feet, hypotonia and, opisthonotouns, hearing impairment, corneal epitheliopathry due to lagophtalmia, pulmonary hypertension, gynecomastia, clitoris/major-minor labia hypertrophy, hyperprolactinemia, dolicocephaly, small low and dysplastic ears, abnormal creases on the forehead, philtrum, and abnormal inner part of eyelids
**Villa (2016)**	t(14;21)(q32.1;p12)-pat	M	4.9y	preterm birth, hypoplastic kidney, cardiac anomaly, thin corpus callosum, polymicrogyria, trigonal cortical heteropia, excarvation of the optic disk, low somatotropic hormones, growth retardation, intellectual disability, frontal bossing, depressed nasal bridge with anteroverted nostrils,, hypoplasia of the zygomatic bones, accentuated and prominent philtrum, macrostomia, macroglossia, and thick and tented upper-lip
**Our case (2018)**	t(14;14)(p11.2;q32.1) *de novo*	F	14y	frontal bossing, widely spaced teeth, broad upper alveolar ridges, broad mouth, downturned corners of the mouth, broad nasal root, deep nasal bridge, carried sparse hair, eyelashes, and eyebrow, growth retardation, growth retardation, and intellectual disability,
	t(14;14)(p11.2;q32.1) *de novo*	M	19y	coarse features with widely spaced teeth, broad mouth, downturned corners of the mouth, broad nasal root, deep nasal bridge, growth retardation, intellectual disability, and right hemiplegia
Pericentric inversion				
**Trunca (1977)**	inv(14)(plq24)-mat	F	2.5y	low birth weight, prominent frontal bossing, poorly differentiated helices, scant eyebrow and eyelashes, mongolid slanting of the palpebral fissures, inverted V-

				shaped upper lip, high arched palate, broad upper alveolar ridge, micrognathia, asymmetry of face , presacral dimple and severe intellectual disability
**Pfeiffer (1978)**	Inv(14)-mat	F	8y	low birth weight, seizure at 6 months, hypotonia, developmental delay, severe intellectual disability, asymmetrical palpebral fissures, ear-flat scapha and prominent antitragus, and finger-long, slender, hyperextensible
**Sliuzas (2008)**	inv(14)(p11.2q32.1)	M	2y	low birth weight, cleft lip and palate, hydrocephaly, nasolacrimal duct stenosis, brachycephaly<br>hyperterolism, wide nipple ,sacral dimple, clinodactyly (5th finger), simian crease (right hand), developmental delay
**Sgardioli (2013)**	inv(14)(p12q31)	F	1y	low birth weight, cardiac anomaly, congenital diaphragmatic hernia, hypothyroidism, gastroesophageal reflux, growth retardation, developmental delay, hypotonia, prominent forehead, flat face, down slanting palpebral fissures, hyperterolism, broad and depressed nasal root and bridge, short nose with anteroverted nares, short philtrum, large mouth, down turned lip, hypoplastic fingers, simgle palmar creases , and umbillical hernia
Tandem duplication				
**Orye (1983)**	tan dep (14) *de novo*	M	6m	growth retardation, flat occiput, hyperterolism, antimongoloid eye slant, sparse hair-eyebrows, eyelashes, bulbous tip of the nose, upturned, prominent upper lip and prominent lower lip, high set nipple, long and slender thumb, overriding 3rd finger by 2nd and 4th finger, hypotonia, and fundus-depigmentation
**Nikolas (1983)**	tan dep (14) *de novo*	F	1m	low birth weight, enlarged fontanelle, hyperterolism, antimongoloid slant, blindness, elongated philtrum, fish-shaped mouth , micrognathia, high arched palate, deep pharynx, malformed and low set ear, umbillical and inguinal hernia, abnormal hands, partial syndactyly (2 and 3 toes), growth retardation, flattened gyri and narrow grooves, and ventricular septal defects
**Carr (1986)**	tan dep (14) parents?	F	29y	preterm birth, frequent infection, growth retardation, skin-tight, hairless (upper and lower limbs), hypertelorism, external ear-poorly developed tragi and helices, large protruding tongue, multiple filled teeth, wide spaced nipples, flexion contractures-elbow, hip, knee joints, cerebral atrophy, and hiatal hernia and reflux (esophagus)
**Madosa (1989)**	tan dep (14) *de novo*	M	1d	low birth weight, frontal bossing, high forehead, overriding cranial suture, prominent epicanthal folds, left eye-coloboma, facial dysmoprhism, deformity of ear, micrognathia, webbing of the posterolateral neck, non-palpable testis, hypospadias,

				imperforated anus,deep sacral dimple, fingerlike thumbs, single transverse palmar creases, 2nd and 4th ties-dorsally displaced, hypotonia, esophageal atresia with tracheoesophageal fistula, mild-thoracic scoliosis, cardiac anomaly, ureteral dilatation, and tortuosity of left ureter
**Gilgenkrant (1990))**	tan dep (14) *de novo*	F	1m	low birth weight, large fontanelle with wide sutures and ossification, nose-thin, parrot-like with anteroverted nostrils, micrognathia, hypoplasia of ossa zygomatia, short philtrum with a tended, prominent upper lip, sparse hair, asymmetric and downslanting palpebral fissures, hypoplastic eyelids, lower lid defects, colobomata of the lt. iris, bilateral slight micropthalmia, lt. embryotoxon, corneal dystrophy, rt. cataract, keratitis, bilateral clinodactyly of the 5th finger, large toes, asymmetric nipples, sacrococcygeal pit, cardiac anomaly, hypotonia, and intellectual disability
**Chen (2005)**	tan dep (14) *de novo*	F	2.5	hyperterolism, epicanthal folds, macrostomia, oligodontia, intellectual disability, hypotonia, microcephaly, high and prominent forehead, blepharophimosis, sparse eyebrow and lashes, broad philtrum, prominent nasal bridge, low-set ear, clinodactyly of the fifth finger, and pyrolic stenosis
**Thiel (2008)**	tan dep (14) *de novo*	F	8y	growth retardation, high forehead, hyperterolism, broad, flat nasal bridge, short philtrum, thin and "cupid bow" upper lip, broad mouth, small mandible, hypoplastic ear helices, tibial deviation of the 4th and 5th toe, sandal gap, mild brachydactyly, clinodactyly, increased joint flexibility of the wrist, developmental delay, and growth hormone deficiency
**Chen (2013)**	tan dep (14) *de novo*	M	6m	normal development until 6 months
Insertion				
**Mikelsaar (1985)**	ins(4;14)(p14;q24q32)-pat	F	9m	low birth weight, dystonia, rigidity, prominent occiput, enlarged anterior fontanelle, narrow palpebral fissures, sparse-hair, eyelashes, eyebrows, micrognathia, and tented upper lip

pat, mat in Rearranged region, paternal and maternal in Rearranged region; d, m, y in Age, day, month, year in Age; NA, not available
